# Supplementary material for: Gut Microbiota Composition in Undernourished Children Associated with Diet and Sociodemographic Factors: A Case–Control Study in Indonesia
Source: Microorganisms. 2022 Aug 30;10(9):1748. doi: 10.3390/microorganisms10091748 (PMC9502830; doi:10.3390/microorganisms10091748)
Supplement: Supplementary file 1 [file microorganisms-10-01748-s001.zip › microorganisms-1847462-supplementary.pdf]

Table S1. Food categories in normal and undernutrition group

Food categories	Group	
	**Normal (n = 20)**	**Undernutrition (n= 20)**
Rice	Rice, porridge, *nasi uduk, lemper, arem-arem,* fried rice and *lontong*	Rice and fried rice
Noodle	Fried chicken, chicken noodle, instant noodles, rice noodles	Instant noodles and chicken noodles
Legume	Oatmeal, *tempe*, *tahu*, peanuts, sweet potato, kacang kulit, green bean porridge, fried sweet potato	*Tempe, tahu,* nuts
Bakery	Bread, biscuit *roma,* dry sponge cake, steamed spone cake, *martabak*, *kue lapis,* donut, *kue pancong*, sandwich	Biscuit, *bakpia*, sweet bread
Egg	Boiled egg, fried egg, scrambled egg, quail egg	Fried egg, boiled egg, salted egg
Fruits	Watermelon, banana, apple, grapes, *duku,* longan	Banana, papaya, watermelon, snake fruit, sapodilla
Vegetables	Sautéed chicory, cap cay, soup, sautéed long beans, bitter melon, cassava leaves, spinach, sauté cauliflower, sautéed eggplant	Soup, young jackfruit, cucumber, *sayur bening,* sautéed pumpkin, water spinach, moringa leaves, sautéed papaya leaves, long beans, banana bud, bean sprouted, sautéed water spinach, spinach
Oil and fat	Palm oil, butter, margarine	Palm oil, vegetable oil
Dairy product	UHT and pasteurized milk, cheese, ice cream, sweet condensed milk	Whole milk, *milo,* UHT milk
Seasoning, spices and food additives	Salt, sugar, soy sauce, ketchup	Salt, sugar, soy sauce, ketchup
Confectionary	Chocolate, candy, strawberry and chocolate jam	Gummy candy, ice cream, chocolate
Seafood	Fried catfish, fried salted fish, fried milkfish, grilled *awu* fish, anchovy, Fried nile tilapia, *pindang* fish, squid, low jawed mackerel	*Pindang* fish, gold fish, *pepes,* mackerel tuna
Meats and poultry	Fried chicken, meatballs, *soto,* grilled sausage, *gulai,* chicken liver, *sate ayam, abon*	*Soto*, fried chicken, chicken intestine
Snacks	Banana chips, *aci* chips, *cilok, cimol, bakwan, chiki,* wafer, popcorn, *taro, molen, nagasari, risoles,* jelly	*Cilok*, wafer, *chiki*, *tic tac*, jelly, *taro*, aci chips, *cimol, cireng, bakwan*
Drinks	Mineral water, orange juice, tea	Mineral water, *es dawet,* syrup, tea, *es lilin,* young coconut water

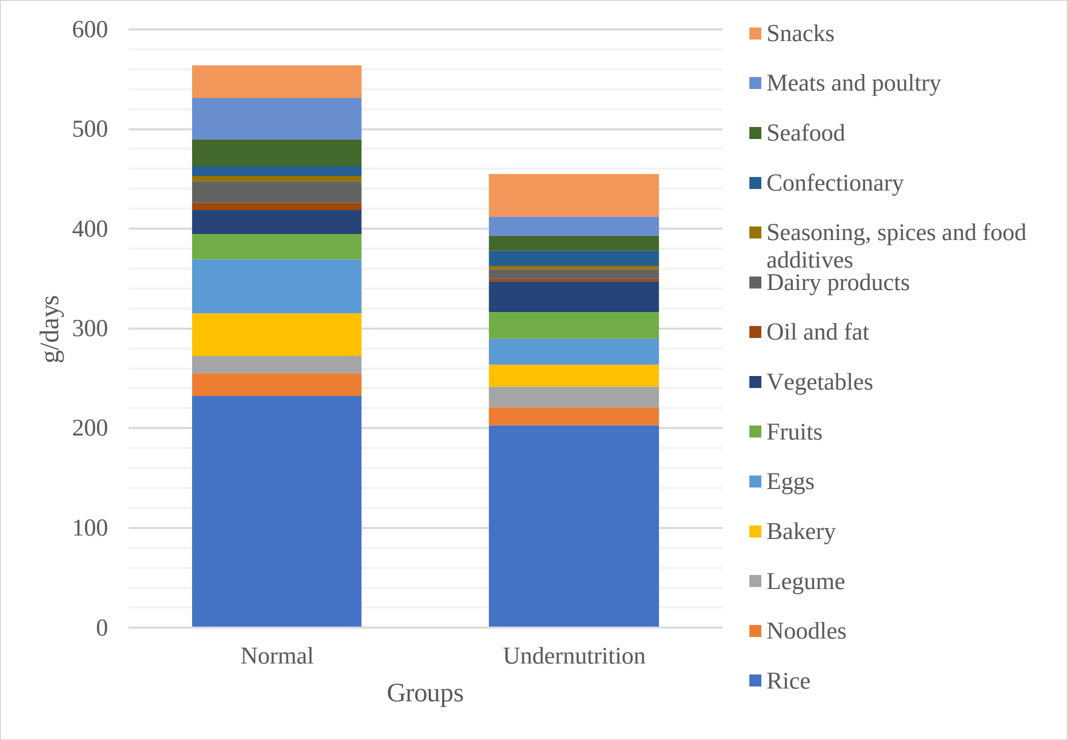

Figure S1. The amount of food consumed each day in normal and undernutrition group
